# Prehospital characteristics of COVID-19 patients in Helsinki – experience of the first wave of the pandemic

**DOI:** 10.1186/s13049-021-00915-0

**Published:** 2021-07-19

**Authors:** Markku Kuisma, Heini Harve-Rytsälä, Jussi Pirneskoski, James Boyd, Mitja Lääperi, Ari Salo, Tuukka Puolakka

**Affiliations:** 1 Departments of Emergency Medicine & Services, Anaesthesiology & Intensive Care Medicine, P.O. Box 112, 00099 Helsinki, Finland; 2 Departments of Emergency Medicine & Services, Helsinki University Hospital and University of Helsinki, Helsinki, Finland

**Keywords:** Emergency medical services, COVID-19, SARS-CoV-2, Ambulance, Prehospital

## Abstract

**Background:**

There is a lack of knowledge how patients with COVID-19 disease differ from patients with similar signs or symptoms (but who will have a diagnosis other than COVID-19) in the prehospital setting. The aim of this study was to compare the characteristics of these two patient groups met by the emergency medical services.

**Methods:**

All prehospital patients after the World Health Organisation (WHO) pandemic declaration 11.3.2020 until 30.6.2020 were recruited for the study. The patients were screened using modified WHO criteria for suspected COVID-19. Data from the electronic prehospital patient reporting system were linked with hospital laboratory results to check the laboratory confirmation for COVID-19. For comparison, we divided the patients into two groups: screening- and laboratory-positive patients with a hospital diagnosis of COVID-19 and screening-positive but laboratory-negative patients who eventually received a different diagnosis in hospital.

**Results:**

A total of 4157 prehospital patients fulfilled the criteria for suspected COVID-19 infection during the study period. Five-hundred-thirty-six (12.9%) of the suspected cases received a laboratory confirmation for COVID-19. The proportion of positive cases in relation to suspected ones peaked during the first 2 weeks after the declaration of the pandemic. In the comparison of laboratory-positive and laboratory-negative cases, there were clinically insignificant differences between the groups in age, tympanic temperature, systolic blood pressure, heart rate, on-scene time, urgency category of the call and mode of transportation. Foreign-language-speakers were overrepresented amongst the positive cases over native language speakers (26,6% vs. 7,4%, *p* < 0,001). The number of cases in which no signs or symptoms of COVID-19 disease were reported, but patients turned out to have a positive test result was 125 (0,3% of the whole EMS patient population and 11,9% of all verified COVID-19 patients encountered by the EMS).

**Conclusions:**

In a sample of suspected COVID-19 patients, the laboratory-positive and laboratory-negative patients were clinically indistinguishable from each other during the prehospital assessment. Foreign-language-speakers had a high likelihood of having Covid-19. The modified WHO criteria still form the basis of screening of suspected COVID-19 patients in the prehospital setting.

## Background

The coronavirus disease 2019 (COVID-19) pandemic has had a tremendous impact on health care systems worldwide. In Finland various recommendations and restrictions for the population and a partial lockdown of the society have helped the health care system to run effectively during the pandemic. The emergency medical service (EMS) is often the first link in the chain of care especially when the symptoms are severe [1–5]. However, the signs and symptoms in COVID-19 are not specific to the disease [6] and they are commonly encountered in several prehospital patient groups. Therefore, only a small portion of all suspected COVID-19 cases met by EMS can be expected to be severe acute respiratory syndrome coronavirus 2 (SARS-CoV-2) positive after laboratory testing.

Prehospital data and hospital records are not usually linked and thus the final proportion of COVID-19 positive patients in the prehospital setting remains unknown. There has also been a lack of knowledge whether the confirmed COVID-19 patients differ clinically from other prehospital patients with similar signs or symptoms. The EMS have been forced to raise the suspicion of COVID-19 with a low threshold if the patient has met the criteria as defined by the World Health Organisation (WHO) [6]. Consequentially, the high number of suspected COVID-19 patients has burdened the emergency departments which may have led to disruption in standard diagnostic protocols and delayed the establishment of differential diagnoses.

Studies of the effects of previous pandemics on EMS are limited. H1N1-pandemic highlighted the need for continued education and communication regarding infection control and the appropriate use of personal protective equipment (PPE) [7]. Refusal of EMS staff to work during a pandemic has been a concern [8]. The challenges in EMS concerning contagious diseases are mainly related to a demanding working environment and to the need for acting rapidly in situations which often have little or no relevant background information of patients.

The aim of this EMS based study was to compare the characteristics of suspected COVID-19 patients who received laboratory confirmation to suspected COVID-19 patients who were laboratory-negative and eventually received a different diagnosis. In addition, we were interested in the clinical features of screening negative (asymptomatic) patients who later received COVID-19 diagnosis.

## Methods

### The study design

We conducted a retrospective cohort study of all patients encountered by the EMS using a uniform electronic patient report (EPR) system.

### The study setting

The capital city of Helsinki and the surrounding area have a population of 1,260,000 inhabitants. The natively spoken languages are Finnish and Swedish. The EMS is organized by Helsinki University Hospital (HUS) and the ambulance services are provided by three Rescue (Fire) Departments and two private ambulance companies. Interhospital patient transportation is not part of EMS. Also, nursing home calls are mainly handled by other agencies than EMS. All emergency phone calls (112) are received and processed by the emergency medical communication centre. The four-step triage classification (A-D) of calls is described in Table 1. EMS has a uniform EPR system (Merlot Medi®, CGI, Finland). Before the pandemic, the annual call volume of EMS was 130,000 (2019). During the first wave of the pandemic the call volume was reduced by 12%,

**Table 1:** The four-step triage classification of the ambulance calls. Class C and D calls are responded to without lights and sirens on and by observing speed limits and regular traffic rules

Triage class	Explanation
A	A high-risk, life threatening situation with a suspected severe disturbance in vital signs or a high energy injury.
B	A situation in which there is a disturbance in vital signs that might progress to be life-threatening without prompt EMS interventions.
C	A situation where patient is stable and can wait for an ambulance. EMS responds within 30 min.
D	A non-urgent situation. EMS responds within 120 min.

### The prehospital COVID-19 screening

The signs and symptoms referring to COVID-19 disease were defined in EMS as: fever ≥38 °C, cough, shortness of breath, sore throat, diarrhoea and/or loss of taste or smell. Any one of these criteria caused patients to be treated as a suspected COVID-19 case. The criteria were slightly more narrow compared to WHO criteria [6] and they were adjusted to EMS patient population (e.g. fatigue alone does not lead to ambulance dispatch in Finland). The emergency medical dispatcher reported the results of their initial COVID-19 screening questions to ambulance crews while they were on their way to the scene. The caller was asked of symptoms of respiratory tract infection, confirmed COVID-19 disease, order to stay in quarantine and contacts with confirmed COVID-19 cases within the previous 14 days. All EMS personnel were instructed to use universal precautions and personal protective equipment in case the patient had any signs or symptoms of COVID-19 disease or the situation was unclear (e.g. patient was unconscious or not able to communicate). Universal masking of personnel and patients in all ambulance calls was not yet used at the time of the study. The EPR system was modified to enable the registering of COVID-19 related information. The data of suspected or already confirmed COVID-19 infection was recorded using structural fields into the EPR system.

### Laboratory testing of SARS-CoV-2

The test used for laboratory confirmation of COVID-19 infection from nasopharyngeal swabs was a real-time reverse transcription-polymerase chain reaction test by the laboratory at the Helsinki University Hospital (HUSLAB COVID-19, Helsinki, Finland). Test results were screened up to 14 days before and 10 day after the EMS contact. Test sampling (nasopharyngeal swabbing) was done only either in hospitals or in outpatient test sites.

### Data collection

**Fig. 1:**
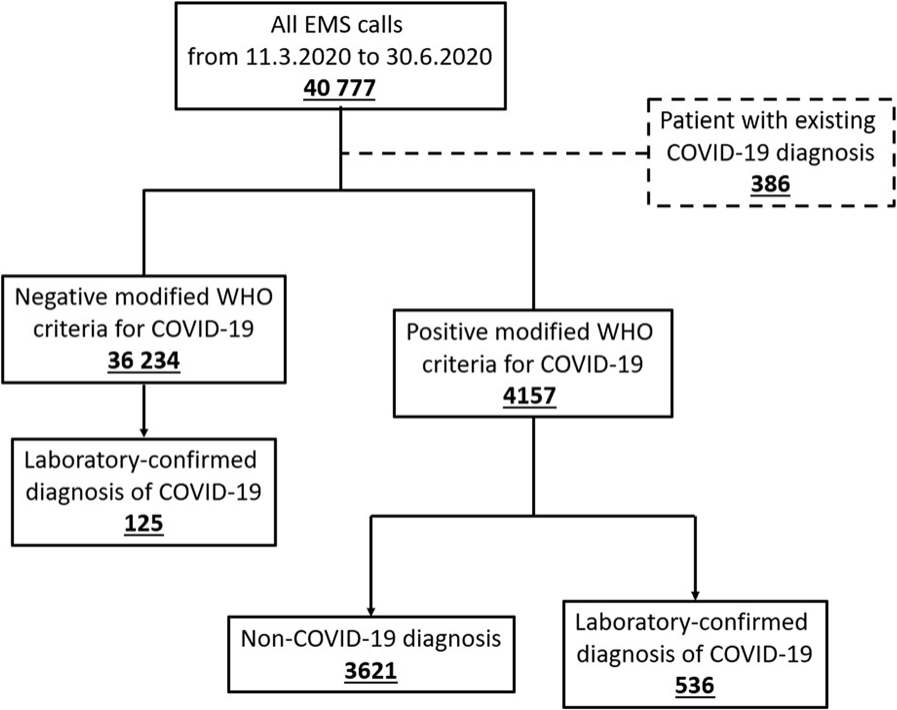
Description of study sample. EMS = emergency medical services

The study period begun from the beginning of WHO pandemic declaration (March 11th, 2020) and continued until the number of new confirmed cases was minimal (June 30th, 2020). This time-period correlates with the first wave of pandemic in Finland. We included all EMS encountered patients in all age groups with a Finnish social security number or a temporary social security number for non-Finnish residents. The exclusion criteria were a diagnosis of COVID-19 at the time of the EMS call (Fig. 1). All prehospital patient reports with suspected COVID-19 infection were retrieved from the EPR system. Patients who already had received a diagnosis of COVID-19 at the time of the EMS call were excluded from the study. The laboratory testing results were retrieved from the hospital laboratory database (Weblab®, Mylab, Tampere, Finland) and combined with prehospital data. For comparison we divided the patients into two groups: screening- and laboratory-positive patients with a diagnosis of COVID-19 and screening-positive but laboratory-negative patients who eventually received a different diagnosis. In addition, a separate subgroup of patients who were screening-negative (asymptomatic) for COVID-19 during the EMS call, but received laboratory-confirmation at the hospital, were examined.

### Statistical analysis

The suspected and confirmed COVID-19 cases were compared using Mann-Whitney U tests and Chi-squared tests. The data are presented using counts, percentages, median and inter-quartile ranges (IQR). *P*-values below 0.05 were considered significant and all tests were two sided. The analyses were done using R version 4.0.3 and the visualizations with the ggplot2 package.

### Ethics

The study plan was approved by the appropriate research body of HUS. Due to the nature of the study neither ethical committee approval nor consent from the patients was required. Finnish research legislation does not require ethical committee approval of retrospective studies based solely on patient records.

## Results

**Fig. 2:**
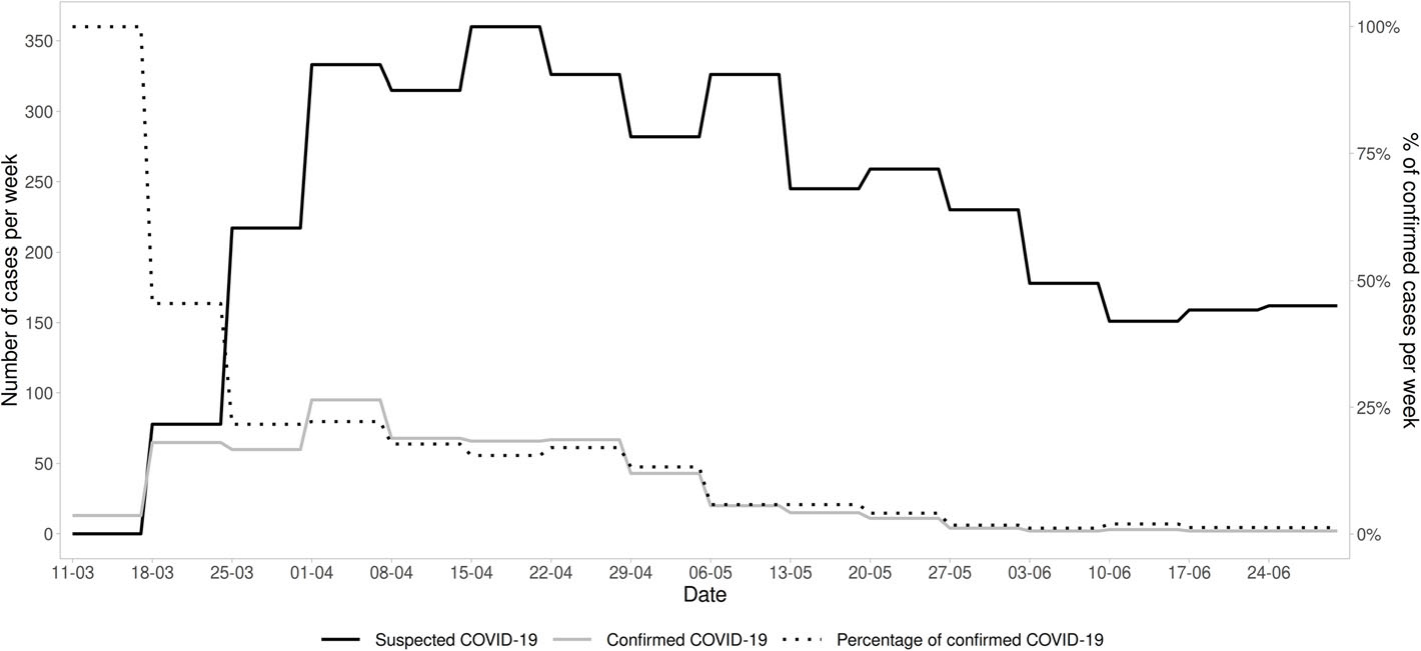
Timeline of suspected cases of COVID-19 with a final diagnosis other than COVID-19 compared against laboratory confirmed COVID-19 cases

During the study period EMS encountered 40,777 patients of which 661 tested positive for SARS-CoV-2 regardless of whether Covid-19 infection was suspected by the EMS staff or not. (Fig. 1). A total of 4157 patients fulfilled the criteria for suspected COVID-19 infection. Five-hundred-thirty-six (12.9%) of the suspected cases received a laboratory confirmation for COVID-19. Additionally, EMS encountered 386 patients with a pre-existing COVID-19 diagnosis who were excluded from the study. The proportion of SARS-CoV-2 positive cases in relation to suspected cases changed drastically during the study period (Fig. 2). The highest proportion of positive cases was seen during the first 2 weeks after the declaration of the pandemic.

### Group comparison

There were statistically significant differences between the groups of suspected and confirmed COVID-19 patients in age, tympanic temperature, systolic blood pressure, heart rate, on-scene time, urgency category of the call, native language of patient and transportation. However, the differences were not clinically meaningful to be used in prehospital assessment. Interestingly, foreign language speakers had a more than three times higher likelihood of having laboratory confirmed Covid-19 than native language (Finnish or Swedish) speakers. All foreign language speakers had Finnish social security number and they were living in Finland. The group comparison is presented in Table 2.

**Table 2:** Characteristics of suspected and laboratory-confirmed cases of COVID-19 (total *n* = 4157). All continuous variables presented in median (interquartile range)

Variable	All patients	Suspected Covid-19	Confirmed Covid-19	***P*** value
Age (years)	72.42 (52.08–82.5)	73.25 (53.83–83.17)	62 (44.83–78.19)	< 0.001
Sex, male (%)	2048 (49.4%)	1789 (49.5%)	259 (48.4%)	0.671
Native language other than Finnish or Swedish (%)	391 (9.8%)	259 (7.4%)	132 (26.6%)	< 0.001
Initial respiratory rate (/min)	20 (16–25)	20 (16–25)	19 (16–24)	0.181
Last respiratory rate (/min)	19 (16–24)	19 (16–24)	18 (16–24)	0.175
Initial blood oxygen saturation (%)	95 (92–97)	95 (92–97)	95 (92–97)	0.444
Last blood oxygen saturation (%)	96 (94–98)	96 (94–98)	95 (93–97)	0.086
Heart rate (/min)	94 (79–110)	94 (79–110)	91 (79–105)	< 0.01
Systolic blood pressure (mmHg)	138 (121–156)	139 (121–158)	133 (120–149)	< 0.001
Temperature (°C)	37.5 (36.9–38.4)	37.5 (36.8–38.3)	37.8 (37.1–38.6)	< 0.001
Blood glucose (mmol/l)	7 (6–8.7)	7.1 (6–8.7)	6.7 (5.8–8.7)	0.129
Urgency of dispatch				< 0.001
*A*	291 (7.0%)	273 (7.5%)	18 (3.4%)	
*B*	891 (21.4%)	797 (22.0%)	94 (17.5%)	
*C*	1250 (30.1%)	1088 (30.0%)	162 (30.2%)	
*D*	1725 (41.5%)	1463 (40.4%)	262 (48.9%)	
On-scene time (min)	25 (19–34)	25 (19–35)	23 (17–30)	< 0.001
Duration of the mission (min)	100 (78–130)	100 (79–128)	99 (72–135.25)	0.219
Transport rate (%)	3498 (84.1%)	3130 (86.4%)	368 (68.7%)	< 0.001

### Asymptomatic COVID-19 patients

The number of cases in which no signs or symptoms of COVID-19 disease were reported in prehospital phase, but patients turned out to have a positive SARS-CoV-2 test result was 125 (0,3% of the whole EMS patient population and 11,9% of all verified COVID-19 patients encountered by the EMS). Characteristics of these patients are described in Table 3. The two leading causes for 112 call were falls and malaise. Atypical causes for requesting an ambulance were mental disturbance (5), drug overdose (4), traffic accident (2) and assault (1). There were no major abnormalities in vital functions of these patients. One patient with fever > 38 °C was missed as being a COVID-19 suspect despite meeting the WHO screening criteria.

**Table 3:** Characteristics of patients with no signs or symptoms of Covid-19 in the prehospital phase but who later tested positive for SARS-CoV-2 at the hospital. All continuous variables are presented using median (interquartile range)

Age, years	75,4 (56,3-84,3)
Sex, male	50 (40%)
Initial respiratory rate (/min)	16 (16–18)
Body temperature (°C)	36,9 (36,5-37,2)
Most common dispatch codes	
Fall	23 (18,4%)
Malaise	23 (18,4%)
Nausea, vomiting and diarrhea	9 (7,2%)
Abdominal pain	9 (7,2%)
Chest pain	7 (5,6%)
Ambulance transport to hospital	90 (72%)

## Discussion

### Principal findings

This is one of the first studies combining comprehensive prehospital EPR data with hospital laboratory-confirmed COVID-19 diagnosis in patients managed by the EMS. We found that the proportion of laboratory confirmed COVID-19 cases in relation to suspected ones was high during the first weeks after the declaration of the pandemic. At that phase patients tended to stay at home too long before seeking diagnostics and treatment. Although several prehospital parameters were associated with the final diagnosis of COVID-19, their clinical significance was low and did not give added value to the modified WHO criteria for a suspect case, except for the native language of the patient.

The modified WHO criteria were negative in 125 patients who later received a laboratory-confirmed COVID-19 diagnosis. Although these cases represented only 0,3% of the whole EMS patient population they cause a potential occupational health risk for EMS personnel. In a study from Seattle, USA, almost one third of COVID-19 patients encountered by the EMS did not present with fever, respiratory difficulty or cough [2].

### Relation of results to the other studies

Most of the previous studies of COVID-19 in the prehospital setting have not focused on patient assessment or diagnostics but rather on demand and performance of the EMS, and occupational safety [1, 3, 5, 9–11]. In a study from Seattle, the EMS had documented the suspicion of COVID-19 in only 50% of cases with an eventual COVID-19 diagnosis [2]. The investigators concluded that the conventional symptoms of febrile respiratory illness may not have the necessary sensitivity for early diagnostic suspicion when screened among EMS patients. It is to be noted that nursing home residents were overrepresented in this study (46% of all COVID-19 patients met by the EMS). Nursing home patients may be more likely to have atypical symptoms compared to those living independently. One of the first published case series on EMS treated COVID-19 infected patients also pointed out this possibility [12].

A recent study from Sweden found that up to 54% of COVID-19 patients presented with primary symptoms not typical of COVID-19 [14]. This proportion was markedly higher than in our study (19%) and it may reflect differences how people with non-severe symptoms use emergency services. Their finding that vital signs had little predictive value in identifying of COVID-19 cases in in accordance with our results. However, deteriorated vital signs may be a predictor of poor outcome in a subgroup of severe cases.

Blood oxygen saturation did not become significant in differentiating the patient groups in our study. However, worsening rates of hypoxemia have been reported in patients with respiratory symptoms treated by EMS after the beginning of pandemic in Tijuana, Mexico [5]. A similar, but more modest finding was reported in Lombardy, Italy [4]. We believe that the good access to health care and availability of EMS in Finland may have caused patients to be encountered before the development of severe hypoxia.

### Relevance of the study results

The study sample included all patients who needed the EMS in the capital area and therefore the results can be considered epidemiologically comprehensive.

In the prehospital setting, initial screening for COVID-19 is mandatory but the key issue is what kind of false negative and false positive screening rates can be accepted. The rates may differ regarding on the point of view – patient assessment or occupational safety of the staff. Patients who do not have, or for some reason do not report, any symptoms in COVID-19 screening are a challenge for EMS. Unexpectedly, we found falls to be a common cause for calling help in this patient group. In elderly people, falls may be caused by medical conditions where the general condition of the patient begins to deteriorate.

If the patient’s native language was other than Finnish or Swedish, we found the likelihood of COVID-19 to be significantly higher. This is supported by the epidemiological data that COVID-19 was more common in certain immigrant groups in Finland. These immigrant families may have several members living in small apartments, which may have important implications for patient assessment and the occupational safety of EMS staff. Our findings are emphasized by the fact that foreign language speakers were not tourists. They were living in Finland permanently or on a long-time basis and they had Finnish social security number.

The ambulance transport rate in the group with a final diagnosis of COVID-19 was lower than in the group with other diagnosis. This finding is not explained by the studied parameters. Some patients were instructed to arrange their own transport to an emergency department. The national recommendation to the public in March “to stay home if you have COVID-19 like symptoms” may have also affected the decision making of EMS personnel.

### Limitations and future studies

The study was limited by the fact that in the early phase of the pandemic, SARS-CoV-2 testing capacity was limited and patients with modest or atypical symptoms may not have been tested as actively as patients presenting during the later phase of the pandemics first wave. In one febrile patient, who later tested positive for SARS-CoV-2, the EMS personnel failed to suspect a COVID-19 infection.

Considering the continuum of this pandemic, future pandemics, and other infectious disease threats, there is a need for studies that can provide tools (e.g. clinical characteristics, triage models [13], point-of-care testing, artificial intelligence) for initial diagnostics of COVID-19 in the prehospital setting.

## Conclusions

Suspected cases of COVID-19 who eventually received a different diagnosis, and laboratory confirmed COVID-19 cases had minor differences in their prehospital clinical parameters which were not clinically significant. Foreign-language-speakers had a high likelihood of having Covid-19. We found no clinical signs that could improve modified WHO criteria in screening for suspected COVID-19 patients in EMS.

## Data Availability

Please contact author for data requests.

## Abbreviations

COVID-19: Coronavirus disease 2019
EMS: Emergency medical services
EPR: Electronic patient report
IQR: Interquartile range
SARS-CoV-2: Severe acute respiratory syndrome coronavirus 2
WHO: World Health Organization
